# Population-based colorectal cancer screening: comparison of two fecal occult blood test

**DOI:** 10.3389/fphar.2013.00175

**Published:** 2014-01-10

**Authors:** Miren B. Zubero, Eunate Arana-Arri, José I. Pijoan, Isabel Portillo, Isabel Idigoras, Antonio López-Urrutia, Ana Samper, Begoña Uranga, Carmen Rodríguez, Luis Bujanda

**Affiliations:** ^1^Clinical Epidemiology Unit, Cruces University Hospital, Basque Health ServiceBarakaldo, Bizkaia, Spain; ^2^BioCruces Health Research InstituteBizkaia, Spain; ^3^Biomedical Research Center Network for Epidemiology and Public HealthBizkaia, Spain; ^4^Colorectal Cancer Screening Programme Coordinating Centre, Basque Health ServiceBizkaia, Spain; ^5^Clinical Biochemistry Service, Cruces University Hospital, Basque Health ServiceBarakaldo, Bizkaia, Spain; ^6^Clinical Biochemistry Service, Donostia University Hospital, Basque Health ServiceDonostia, Gipuzkoa, Spain; ^7^Digestive Department, Donostia University Hospital, Basque Health ServiceDonostia, Gipuzkoa, Spain; ^8^Clinical Biochemistry Service, Araba University Hospital, Basque Health ServiceGasteiz, Araba, Spain; ^9^Biodonostia Research InstituteDonostia, Spain

**Keywords:** immunochemical fecal occult blood test, population screening, health outcomes research, health impact assessment, colorectal cancer

## Abstract

**Background:** The aim of screening for colorectal cancer is to improve prognosis by the detection of cancer at its early stages. In order to inform the decision on the specific test to be used in the population-based program in the Basque Autonomous Region (Spain), we compared two immunochemical fecal occult blood quantitative tests (I-FOBT).

**Methods:** Residents of selected study areas, aged 50–69 years, were invited to participate in the screening. Two tests based on latex agglutination (OC-Sensor and FOB Gold) were randomly assigned to different study areas. A colonoscopy was offered to patients with a positive test result. The cut-off point used to classify a result as positive, according to manufacturer’s recommendations, was 100 ng/ml for both tests.

**Results:** The invited population included 37,999 individuals. Participation rates were 61.8% (*n* = 11,162) for OC-Sensor and 59.1% (*n* = 11,786) for FOB Gold (*p* = 0.008). Positive rate for OC-Sensor was 6.6% (*n* = 737) and 8.5% (*n* = 1,002) for FOB Gold (*p* < 0.0001). Error rates were higher for FOB gold (2.3%) than for OC-Sensor (0.2%; *p* < 0.0001). Predictive positive value (PPV) for total malignant and premalignant lesions was 62.4% for OC-Sensor and 58.9% for FOB Gold (*p* = 0.137), respectively.

**Conclusion:** OC-Sensor test appears to be superior for I-FOBT-based colorectal cancer screening, given its acceptance, ease of use, associated small number of errors and its screening accuracy. FOB Gold on the other hand, has higher rate of positive values, with more colonoscopies performed, it shows higher detection incidence rates, but involves more false positives.

## INTRODUCTION

Colorectal cancer (CRC) is the third leading cancer and the fourth leading cause of cancer deaths worldwide, with 1.2 million estimated new cases and 609,000 estimated deaths in 2008 (Karsa et al., 2010). In the European Union (EU), as well as in Spain, CRC is the third leading newly diagnosed cancer, after lung cancer and prostate cancer in males and the second after breast cancer in females and the second leading cause of overall cancer deaths (Karim-Kos et al., 2008). Due to its high frequency, mortality and morbidity rates, and the high socio-economic burden associated with this disease, CRC has become an important and challenging public health problem (Karsa et al., 2010).

In the Basque Country (one of 17 Autonomous Regions of Spain), once age-adjusted at European standard population, CRC incidence (86.37/100,000 among men and 39.75/100,000 among women in 2009), and mortality rates (20.6/100,000 in 2009), have shown moderate increases, mainly in men from 1986 to 2008 (Izarzugaza et al., 2010).

Nowadays, a substantial amount of information is available on CRC etiology and prognosis. Its slow growth from benign lesions is currently well known and this makes many of these lesions detectable and removable at an early stage (Scottish Intercollegiate Guidelines Network [SIGN], 2011).

Primary prevention is based mainly on the adoption of healthy lifestyle measures including changes in dietary habits (Kahi et al., 2008). However, the benefits of both healthy lifestyle measures become visible in the long-term. It is therefore necessary to design and implement programs that allow early detection and management of pre-cancerous and cancer lesions (secondary prevention; von Karsa et al., 2008).

The new European Code against Cancer (2003/878/EC; European Code Against Cancer, 2012) includes among its recommendations that “men and women from 50 years of age and older should participate in CRC screening.” Both the EU and the Comprehensive Cancer Plan of the Spanish Ministry of Health, Social Services and Equity (2006; Ministerio de Sanidad y Política Social, 2010) include the implementation of screening programs among their recommendations for CRC prevention. Nationwide CRC screening programs are currently being implemented in several European countries as well as Spain. Effective screening methods have been shown to decrease CRC incidence rates by 20% and mortality rates by 33% (Mandel et al., 1993; Shaukat et al., 2013).

Up to now, the guaiac fecal occult blood test (g-FOBT) was considered the standard screening test used in CRC detection programs. However, recently marketed immunochemical tests (I-FOBT) have become widely accepted due to several advantages over g-FOBT (Rozen et al., 2009; Oono et al., 2010), and its use is recommended for population-based programs (Segnan et al., 2011). Advantages include a higher sensitivity, specificity for human hemoglobin (Allison et al., 2007), fewer stool samples required and no diet or medications restrictions are needed. Additionally, the quantitative nature of I-FOBT results allows for an optimal cut-off point to be set for a nationwide screening program (Castiglione et al., 2002; Wong et al., 2003; Fraser et al., 2006; Guittet et al., 2007; Levi et al., 2007), based on pre-specified criteria.

Adherence rate is of paramount importance to ensure CRC screening programs effectiveness. In this sense, immunochemical tests show higher adherence (Cole et al., 2003; Van Rossum et al., 2008; Hol et al., 2010) and detection rates (Smith et al., 2006; Guittet et al., 2007; Van Rossum et al., 2008) than g-FOBT.

The I-FOBT testing samples can be analyzed automatically, which involves important advantages in terms of quality assurance and costs (Levi et al., 2007).

The Basque Ministry of Health approved in 2008 the implementation of a population-based screening program through the detection of fecal occult blood (FOB) using I-FOBT as the screening method every 2 years and colonoscopy as a confirmation test. An important issue to be dealt with was decision on the particular I-FOBT to be used as two commercial diagnostic kits were available in the Spanish market at the time screening activities were about to start.

A comprehensive literature search did not yield consistent information regarding comparison of analytical and operational characteristics among marketed immunochemical tests. Just one journal article addressing the compared diagnostic efficacy of two different I-FOBTs was found, with no definitive results (Rubeca et al., 2006).

Therefore, we aimed to compare the two available I-FOBT tests: OC-Sensor (Eiken Chemical Co., Tokyo, Japan) and FOB Gold (Sentinel Diagnostics SpA, Milan, Italy) in terms of diagnostic performance, ease of use, acceptance, and operational features within the context of the pilot phase of a CRC population-based screening program. The outcomes of that study would then inform the decision-making process to choose the screening test for the ulterior full implementation.

## METHODS

This study involved the first round of a CRC population-based screening program in the Basque Country – restricted to some previously determined health districts – from January 2009 to March 2010, and invited residents aged 50–69, from the Basque Health Service database (*n* = 37,999). People with colorectal cancer resection (CCR) history and who had undergone a previous colonoscopy within the past 5 years were excluded.

Participation was voluntary and was offered to all subjects residents who lived in the areas designated for the study and had a general practitioner (GP) assigned.

The Screening Management Centre sent a letter explaining the aims and methods of the screening program to all eligible subjects. After 7–10 days, a second letter was sent with a request for them to participate in the program, including a kit package specifically suited to collect a single sample of feces, and stickers with the uptake’s data to be attached to the tube. Participants could leave the kit sample at the Primary Care Centers during working hours (from 8:00 am to 8:00 pm). Samples were processed by trained laboratory staff following the instructions provided by the manufacturers. At health-district level, neither health professionals nor administrative staffs were aware that a comparison of screening kit tests was being conducted. Each assay test was randomly assigned by an independent researcher to each of five health districts where the program was being implemented. According to both manufacturer’s instructions, tests were considered positive when the sample contained at least 100 ng/ml of hemoglobin by buffer. A colonoscopy was offered to all positive participants by their GP. When errors were identified by any laboratory before or after analyzing the sample, a new kit was sent to the participant and the new sample analyzed. True positives were defined by colonoscopy examination and pathology analysis. CRC screening performance measures were assessed following the National Guidelines published in 2009 (Castells et al., 2009). Every case was codified by expert staff in the Screening Management Centre. Advanced adenoma was considered: >10 mm, or 3–10 adenomas or villous morphology, or high degree of dysplasia. Cancer colorectal was considered pT1.

This study was conducted under real practice conditions, which is why we did not perform colonoscopies for patients with a negative FOBT test. To control the false negatives interval cancers are followed, as recommended by the European Guidelines (von Karsa et al., 2008).

The study was submitted and approved by the Ethics Committees of screened areas.

### STATISTICAL ANALYSIS

Colorectal cancer screening performance measures were assessed following the European Guidelines (Segnan et al., 2011). Chi-square tests were used to compare proportions between relevant subgroups. Log-binomial regression models (Barros and Hirakata, 2003) were fitted to yield age and sex-adjusted comparisons among assessed kits, in terms of corresponding participation rates, positive predictive values, error rates, and cancer and advanced adenomas incidence rates. In order to assess magnitude and statistical significance of the effects of predictors of interest, average marginal effects (AME; mean change in predicted probabilities of the response variable across all sampled individuals when the categorical predictor changes by one level with respect to the reference level, keeping all other predictors at observed values) were calculated (Bartus, 2005). In some instances, to provide additional information on the magnitude of effects, relative estimates are given in the form of relative risks (RR) as estimates of prevalence ratios. Standard errors that took the cluster (health district assignment of kits) structure of the data into account were estimated using the delta method. Estimated models included statistically significant interactions. As gender–age group interactions were most often encountered, AME are shown by gender and age stratum combinations to ease interpretation of effects. Significance level was set to 5%.

Statistical analyses were performed using Stata 12 for Windows (StataCorp. 2011. Stata Statistical Software: Release 12. College Station, TX: StataCorp LP).

## RESULTS

### PROGRAM COVERAGE

The target population included 39,566 individuals. Finally, a total of 37,999 individuals aged 50–69 and who met the participation criteria, were invited to participate. **Table 1** shows the characteristics of this population and performed procedures. Statistically significant differences in age distribution were found between invited individuals assigned to the study kits [mean age for population receiving FOB Gold 58.6 years (SD: 5.6) and 59.1 (5.8) for population receiving OC-Sensor; *p* = 0.000]. The same pattern of overall and strata-based age differences was found between individuals of both participants groups [mean age for FOB Gold 58.9 (5.5) and 59.5 (5.7) for OC-Sensor]. No statistically significant differences were found in gender distribution between invited people (50.2% of people receiving FOB Gold test were women vs. 50.8% in population receiving OC-sensor; *p* = 0.233).

**Table 1:** Invited population, age, and sex distribution of participants and tests performed as a result of the screening program.

	OC-Sensor		FOB Gold		Overall	
	Mean	SD	Mean	SD	Mean	SD
Age (year) of invited individuals	59.1	5.8	58.6	5.6	58.8	5.7

	*n*	%	*n*	%	*n*	%

Invited population	18,064	100	19,935	100	37,999	100
Participants	11,162	61.8	11,786	59.1	22,948	60.4
Age distribution (year)						
50–54	2729	24.5	3163	26.8	5892	25.7
55–59	2711	24.3	3156	26.8	5867	25.6
60–64	3096	27.7	3169	26.9	6265	27.3
65–69	2626	23.5	2298	19.5	4924	21.4
Gender						
Male	5187	46.5	5529	46.9	10716	46.7
Female	5975	53.5	6257	53.1	12232	53.3
I-FOBT results						
Positive	737	6.6	1,002	8.5	1,739	7.6
Negative	10,416	93.3	10,723	91.0	21,139	92.1
Result not available	9	0.1	61	0.5	70	0.3
Patients with colonoscopy performed	686	93.1^1^	914	91.2^1^	1,600	92.0^1^
Colonoscopic result available	686	93.1^1^	912	91.0^1^	1,598^2^	91.9^1^

1 Percentages of participants with the I-FOBT test positive;

2 1,576 colonoscopies with conclusive results.

Overall participation rate was 60.4% (OC-Sensor assay 61.8% vs. 59.1% for the FOB Gold, *p* = 0.008). It was consistently and significantly higher for females with an overall marginal effect estimate of 6.8% increase in participation rate relative to men (**Tables 2** and **3**). Participation rate with OC-Sensor test was higher for both sexes (65.1 vs. 62.6% in women and 58.3 vs. 55.6% in men) and kept consistently higher (range of differences: 1.5–3.5%) across all age groups. However, this difference was not statistically significant when the cluster-randomized design was accounted for in the analysis (RR = 1.05; 95% CI = 0.93–1.18) Participation rates increased significantly with age in both groups, showing a gradient which reached its highest value in those aged 60–64. An interaction between gender and age was found in the two oldest strata, reflecting a larger increase in rates among men with stabilization in the oldest strata and a smaller increase among women aged 60–64 with a moderate reduction in the oldest age group. Estimates of marginal effects on participation are, hence, presented separately by gender (**Table 3**).

**Table 2:** Results of participation rates and true positive rates by gender, age, and type of kit administered (true positive rates based on 1,576 colonoscopies performed with conclusive results).

	Female			Male		
	OC-Sensor	FOB Gold	Total	OC-Sensor	FOB Gold	Total
**Participation rates (%)**
Total	65.1	62.6	63.8	58.3	55.6	56.9
**Age (year)**
50–54	59.5	57.8	58.5	51.0	49.7	50.3
55–59	67.0	63.6	65.2	57.6	55.6	56.5
60–64	69.3	67.2	68.2	63.4	59.9	61.5
65–69	64.9	62.5	63.8	62.8	59.3	61.1
**True positive rates (%)**
Total	35.2	31.0	32.8	54.3	49.1	51.3
**Age (year)**
50–54	36.8	32.9	34.2	41.7	36.5	38.3
55–59	33.3	29.6	31.3	52.3	44.5	48.0
60–64	35.4	31.3	32.9	56.9	53.1	54.8
65–69	35.8	30.2	32.9	59.2	57.5	58.3

**Table 3:** Influence of the type of kit administered, gender and age on uptake and true positive rates.

Participation rates (%)						
Factor	AME	CI	*p*-Value			
Female	6,8	(5.9/7.6)	0.000			

**Males**				**Females**		
**Factor**	**AME**	**CI**	***p*-Value**	**AME**	**CI**	***p*-Value**

Kit	2.6	(-5.1/10.2)	0.513	2.4	(-4.8/9.6)	0.512
Age (55–59)	6.2	(3.8/8.7)	0.000	6.7	(5.9/13.3)	0.000
Age (60–64)	11.2	(8.5/13.9)	0.000	9.6	(5.9/13.3)	0.000
Age (65–69)	10.7	(6.2/15.1)	0.000	5.1	(1.9/8.3)	0.002

**True positive rates**						
**Factor**	**AME**	**CI**	***p*-Value**			
Female	-18.3	(-22.1/-14.6)	0.000			

**Males**				**Females**		
**Factor**	**AME**	**CI**	***p*-Value**	**AME**	**CI**	***p*-Value**

Kit	4.1	(-0.4/8.6)	0.074	4.4	(-4.9/13.8)	0.354
Age (55–59)	9.4	(3.5/15.4)	0.002	-3.5	(-11.7/4.7)	0.402
Age (60–64)	16.1	(11.7/20.6)	0.000	-1.7	(-3.6/0.3)	0.096
Age (65–69)	19.6	(11.4/27.7)	0.000	-2.0	(-12.8/8.9)	0.721

*
Results are presented separately for men and women as there was evidence of interaction between gender and age. AME, average marginal effects; CI, 95% confidence intervals. Reference groups are males and 50–54 years age group.*

### OUTCOMES WITH FECAL OCCULT BLOOD TESTING

Positive rate for OC-sensor was 6.6 and 8.5% for FOB Gold (RR = 0.77; 95% CI = 0.69–0.87; *p* = 0.000). FOB Gold had consistently higher positive rates than OC-Sensor across all age and sex strata, except for women in the 54–59 year age-group, where rates were very similar (OC-Sensor 4.6 vs. 4.5% for FOB Gold).

True positive rates were higher for OC-Sensor across all age-sex strata (**Table 2**). Due to age–gender interaction, results are again shown separately for men and women. Among men this difference among kits was statistically significant on the relative scale (RR = 1.07; 95% CI = 1.00–1.14) and marginally significant on absolute scale (**Table 3**). Among women differences were not statistically significant on either scale (RR = 1.04; 95% CI = 0.84–1.29). Increasing age was significantly associated with substantially higher positive rates only among men (**Table 3**).

Significant differences were found between the compared kits in relation to the total number of errors [error rate for OC-Sensor 0.24% and for FOB Gold 2.35%, AME: -2.1 (-0.2/-4.0)]. Being female was marginally associated to a lower error rate [RR = 0.85 (0.72–1.01), *p* = 0.06]. Increasing age was also associated to a higher error rate. Again there was interaction between age and sex, with the highest risk in men of 60–64 years of age [RR = 1.62 (1.36–1.92)] and in women of the 65–69 years stratum [RR = 2.14 (1.71–2.67)]. Most of the errors found in the case of FOB Gold were produced as a result of an incorrect sample manipulation by the participants.

### OUTCOMES WITH COLONOSCOPY

The results of the colonoscopies are shown in Table **4.** No significant differences were observed between OC-Sensor and FOB Gold groups. Although 80% of cancers detected in the OC-Sensor group were early cancers (stages I–II) vs. 56.8% in the FOB Gold group, due to the small number of malignancies detected, this difference was not statistically significant.

**Table 4:** Results of the colonoscopies and stage distribution of diagnosed cancers.

	OC-Sensor			FOB Gold		
	*n*	%	95% CI	*n*	%	95% CI	*p-value*
Normal	201	29.3	25.8–32.8	302	33.1	30.1–36.3	n.s
Polyp	39	5.7	4.0–7.7	52	5.7	4.3–7.4	n.s
Not advanced adenoma	106	15.4	12.8–18.3	156	17.1	14.7–19.7	n.s
Advanced adenoma	282	41.1	37.4–44.9	338	37.1	33.9–40.3	n.s
Cancer*	35	5.1	3.6–7.0	44	4.8	3.6–6.5	n.s
Stage I	18	51.4	35.1–67.5	17	38.6	24.4–54.5	n.s
Stage II	10	28.6	15.5–45.0	8	18.2	8.2–32.7	n.s
Stage III	6	17.1	7.2–32.3	13	29.5	16.8–45.2	n.s
Stage IV	1	2.9	0.7–14.9	5	11.4	3.8–24.6	n.s
Unknown	0	0	0–10.0	1	2.3	0.06–12.0	n.s
Non-neoplasic pathology	8	1.2	0.5–2.2	9	1.0	0.4–1.9	n.s
Inconclusive	15	2.2	1.2–3.6	11	1.2	0.6–2.1	n.s
Total	686	100	–	912	100	–	

n.s., non-significant. *Percentages for stages of cancer are based on the total number of cancers instead of total number of colonoscopies.

**Table 5** shows the detection rates of advanced adenomas and cancerous lesions among screening participants. FOB Gold assay users showed higher rates overall and in most age-sex strata. Statistically significant interactions were found between type of kit and gender with age strata and, in order to ease interpretation of results, marginal predicted rates are used. Differences in marginal predictions were highly statistically significant according to type of kit assay (*p* = 0.000) with almost 10 more lesions detected with FOB Gold per 1,000 participants as compared to OC-Sensor. Being female was also strongly associated with a lower detection of pre-malignant and malignant lesions (*p* = 0.000) whereas increasing age was significantly associated with consistently higher detection rates (*p* = 0.000; **Table 5**).

**Table 5:** (a) advanced adenoma and (b) cancer detection incidence rates according to gender, age group, and kit assay; (c) estimated marginal predictions of advanced adenomas and cancer detection rates according to gender, age group, and kit assay (based on health-district adjusted log binomial regression model including interactions between kit and gender with age group).

	Female			Male		
	OC-Sensor	FOB Gold	Total	OC-Sensor	FOB Gold	Total
**(a) Advanced adenoma detection rates (‰)**
Total	12.6	14.3	13.4	39.8	45.1	42.5
**Age (year)**
50–54	8.3	12.9	10.8	18.6	35.9	21.5
55–59	13.1	11.9	12.5	44.6	35.9	39.9
60–64	11.9	14.8	13.0	45.3	55.6	50.5
65–69	17.2	18.8	17.9	51.0	70.7	60.4
**(b) Cancer detection rates (‰)**
Total	2.0	2.4	2.2	4.4	5.2	4.8
**Age (year)**
50–54	1.4	2.9	2.2	0.8	2.7	1.8
55–59	1.4	0.6	0.9	0.8	5.4	3.3
60–64	1.8	3.0	2.4	7.1	2.7	4.8
65–69	3.6	3.4	3.5	8.9	11.6	10.2
**(c) Marginal predicted detection rates (‰)**
**Gender**	**Predicted**	**95% CI**				
Male	68.4	64.8–72.0				
Female	40.8	37.9–43.7				
**Age (year)**
50–54	28.4	26.5–30.3				
55–59	43.2	37.7–48.7				
60–64	50.4	46.6–54.3				
65–69	64.0	57.8–70.1				
**Kit**
FOB Gold	50.6	49.0–52.3				
OC-Sensor	40.8	37.9–43.8				

## DISCUSSION

When a population-based screening program is to be implemented, one key issue to deal with is selection of the screening test to use. In the Basque Country initial decision considered that the population-based CRC screening program was to be based on I-FOBT. Accordingly, in the context of a progressive implementation of the CRC population-based program, a quasi-experimental study has been carried out aimed to compare the diagnostic accuracy and operational characteristics of the two available marketed I-FOBT tests, FOB Gold and OC-Sensor. Manufacturers’ recommended cut-off levels have been used (Vilkin et al., 2005; Rubeca et al., 2006; Levi et al., 2007). In our study, several remarkable differences have been found between the diagnostic kits compared. It may be possibly attributable to differences in the quantity of buffer and other features (NHS Purchasing and Supply Agency, 2009; Moss et al., 2010).

Overall participation rate (60.4%) in this pilot program is well above the minimum acceptable recommended (Segnan et al., 2011) and similar or higher than reported rates in other pilot or established screening programs (UK Colorectal Cancer Screening Pilot Group, 2004; Department of Health, 2006; Málaga López et al., 2010). This may be partly attributed to the use of an invitation approach based on mail contact with the target population that included sending the fecal sampling kit (Van Roosbroeck et al., 2012). Participation rates were almost 7% higher in women and increased with aging in both sexes reaching a peak in the 60–64 age group. This result is in agreement with most but not all FOBT-based screening programs conducted in Europe and Australia (Von Euler-Chelpin et al., 2010; Australian Institute of Health and Welfare, 2012). There was a significant decline in participation among women of the oldest group, but not among men. Other papers have reported a drop in participation rates in oldest age groups as well (Von Euler-Chelpin et al., 2010). This finding cannot be compared with results from the single published randomized comparison of diagnostic performance of both tests (Rubeca et al., 2006) as participation required using both diagnostic kits simultaneously. However, one of the main strengths of our study is that it was conducted in standard of care conditions.

The use of OC-Sensor assay resulted in a consistently increased but not statistically significant absolute participation rates of around 2–3% which might have actual practical relevance though. Gender and age-related participation patterns were similar for both assays. As we did not survey participants or qualitatively analyzed on individual characteristics or potentially relevant operational issues such as ease of use of received kits or other factors, we cannot make any conclusive statement about the reasons for this observed differences. Rates of positive tests were higher than referred in most screening programs that employed any of these tests with 100 ng/ml as cut-off level on average-risk individuals (Castiglione et al., 2002; Rubeca et al., 2006). Rates were significantly higher among FOB Gold users which resulted in this group undertaking an increased amount of colonoscopies (26% increases) compared to OC-Sensor users. These results are in conflict with the work by Rubeca et al. (2006) which found slightly higher positive diagnostic rates among OC-Sensor users. Several screening strategies have established different cut-off points for positive results using OC-Sensor assay (Van Rossum et al., 2009; Wilschut et al., 2011; Faivre et al., 2012), 50–75 ng/ml, but in our population, based on observed positivity rates, lowering the cut-off may not be appropriate without careful consideration of the amount of extra resources (colonoscopy and pathology procedures) involved and the iatrogenic consequences of false positives.

With regard to true positive rates (positive predictive values), several results are remarkable. First of all, true positive rates were much higher among men across all age strata. Secondly the use of OC-Sensor assay was associated with higher rates. The higher percentage of participation found among women when compared to men across all age levels is according with other studies (UK Colorectal Cancer Screening Pilot Group, 2004; Department of Health, 2006; Málaga López et al., 2010; Moss et al., 2010). Participation with OC-Sensor was higher than with FOB Gold, for both sexes and across all age groups, but this differences were not statistically significant when the health-district unit of assignment was considered in the analysis.

With respect to the relationship between age and participation, most studies indicated an inverted “U” shaped function with lowest rates of participation in 50–55 years old and those 70–80 (Australian Institute of Health and Welfare, 2012; Faivre et al., 2012). Our results, although do not include individuals 70 years of age and older, seem to be in agreement with this functional relationship.

A differential gender pattern of true positive responses was found. Whereas among women neither age nor the kit utilized influenced the probability that a positive result was in fact due to a premalignant or malignant lesion detectable by colonoscopy, among men increasing age and the use of OC-Sensor kit were associated to a higher prevalence of true positives.

We have also observed that gender and age are related to differences in the detection rates of advanced adenomas and cancer; with higher rates in men and higher rates by age group. We have observed that in our population detections rates of adenomas are higher than in other studies (Bartus, 2005; Vilkin et al., 2005; Smith et al., 2006). When we analyze the results by type of kit with FOB Gold we can conclude that: on the one hand, it has higher rate of positive values, with more colonoscopies performed, and on the other hand, it shows higher detection rate but involves more false positives.

We believe the strengths of this study include quasi-experimental design, comparison of two I-FOBT tests following manufacturer’s recommendations.

Possible Limitations of the study: (i) cluster randomized design with small number of clusters leading to baseline imbalance (but we have used analytical techniques that take clustering effect into consideration) (ii) lack of measurement on potentially important covariates either at individual level (socioeconomic level, education, etc.) or at cluster level (deprivation index, ethnic distribution, etc.). As a result there might be important predictors confounding the estimates of effect of the type of kit used. The characteristics of the baseline population in the Basque Country (homogeneity) and the randomization of the assignment of the kits could counterbalance the effects of this lack of measurement.

## CONCLUSION

OC-Sensor test appears to be superior for I-FOBT-based CRC screening, given its acceptance, ease of use, associated small number of errors and its screening accuracy. The goal of screening programs is the early detection and removal of neoplasms and, above all, the secondary prevention of colorectal cancer in the general population. Although the interval cancer period is required to establish a proper comparison, the advantages found in this analysis are consistent and lead to the selection of OC sensor as the kit to be used in the CRC population-based program in our region.

## Conflict of Interest Statement

The authors declare that the research was conducted in the absence of any commercial or financial relationships that could be construed as a potential conflict of interest.
